# A classification model for municipalities in the paraense Amazon regarding the risk of violence against women: A multicriteria approach

**DOI:** 10.1371/journal.pone.0292323

**Published:** 2023-10-23

**Authors:** João Lúcio de Souza, Saulo William da Silva Costa, Fernando Augusto Ribeiro Costa, Alana Miranda Medeiros, Guilherme N. DeSouza, Marcos César da Rocha Seruffo

**Affiliations:** 1 Graduate Program in Electrical Engineering, Institute of Technology, Federal University of Pará, Belém, PA, Brazil; 2 Federal Institute of Education, Science and Technology of Pará, Campus Óbidos, Óbidos, PA, Brazil; 3 Graduate Program in Sustainable Development of the Humid Tropics, Center for Advanced Amazonian Studies, Federal University of Pará, Belém, PA, Brazil; 4 Graduate Program in Computer Science, Institute of Exact and Natural Sciences, Federal University of Pará, Belém, PA, Brazil; 5 Operational Research Laboratory-LPO, Institute of Technology, Federal University of Pará, Belém, PA, Brazil; 6 Dept. of Electrical Engineering and Computer Science (EECS), University of Missouri-Columbia, Columbia, Missouri, EUA; Friedrich-Alexander-Universität Erlangen-Nürnberg: Friedrich-Alexander-Universitat Erlangen-Nurnberg, GERMANY

## Abstract

Violence against women (VAW) is a serious violation of the rights to life, health, and physical integrity. Recent studies point out that social, economic, and demographic factors directly impact the advance of this type of violence. In view of these facts, the state has its responsibility increased when it cannot provide the public equipment necessary for management strategies that collaborate with the confrontation of violence. This project aims to develop a multicriteria decision analysis model (MCDA) to classify Pará municipalities with regard to the propensity for VAW crime, based on the mapping of assistance and protection equipment, as well as socioeconomic indicators of each municipality. The model developed and the research findings represent an important step in elaboration. In turn, this model demonstrates its ability to be a possible instrument that decision makers and implementers of public policies aimed at protecting and supporting women victims of violence in order to anticipate new occurrences.

## 1 Introduction

In 2023 it will be three decades since the United Nations signed the Vienna Declaration at a conference in which the horizons of understanding and the scope of what was considered human rights were significantly expanded. Therefore, the international community considered violence against women (VAW), privately or in public spaces, and in any respect, to be incompatible with human dignity, and the rights of women were declared inalienable, integral, and indivisible from the set of universal human rights [1]. Despite this, the recent COVID-19 pandemic showed that there is still much to be done to prevent, combat and punish VAW, as well as to help and protect victims, since the increase in cases was seen as a deleterious consequence of domestic confinement [2, 3].

In a recent study, [4] notes that research on domestic violence has grown over time, with a significant increase in the number of publications in recent decades. However, the authors also identifies limitations in research, such as the lack of reliable data in many countries and the under reporting of domestic violence. These findings highlight the need for continued research on domestic violence and to address these issues to improve our understanding of domestic violence and develop effective interventions.

There are several factors that can play a role in analyzing the cases of VAW between intimate partners. According to the research by [5], there are factors that may be preponderant when analyzing the cases of VAW of the intimate partner. Some of these factors include: the age of the male partner, level of education, socioeconomic status, religiosity, exposure to domestic violence in childhood, acceptance of traditional gender norms and belief in male superiority.

In the context of interpersonal relationships, a dark side emerges that transcends the physical and emotional sphere: economic violence. This manifestation of control and power, as defined by [6], is aimed at creating economic dependence in the victim, by taking control of their financial resources. According to the author, economic violence involves controlling a woman’s ability to obtain, use, and maintain economic resources, which threatens her economic security and potential for self-sufficiency.

Violence against women (VAW) can be defined as any act of gender-based violence that results in or is likely to result in physical, sexual, or mental harm or suffering to women [3, 7]. This type of violence causes physical, material and mental harm to the victims, forcing the application of specific laws to repress it, such as, for example, in Brazil, the Federal Law No. 11.340/2006 (http://www.planalto.gov.br/ccivil_03/_ato2004-2006/2006/lei/l11340.htm), known as Maria da Penha Law, which created mechanisms to curb domestic and family violence against women, ensuring the creation of specific courts and punishments for aggressors in order to eradicate and prevent this type of rights violation whose end point, sometimes, is the victim’s death.

**Fig 1:**
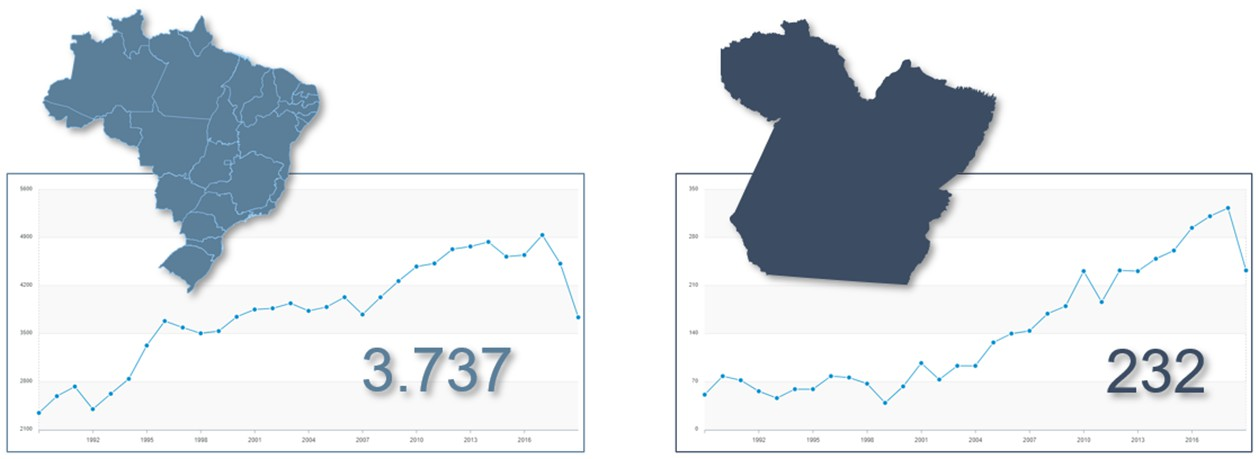
Evolution of the homicide rate per 100,000 female inhabitants in the State of Pará and Brazil. This figure was prepared based on data from table 5.1 of the 2021 atlas of violence. [8]. The underlying basemap is derived from ‘Natural Earth’ global vector data (https://www.naturalearthdata.com).

Thus, the Atlas of Violence, organized by the Institute for Applied Economic Research (IPEA, in Portuguese) [8], 2019, points out that in Brazil there has been a growth in the cases of homicides of women in recent years, reaching a peak of 4,936 cases in 2017, reducing in subsequent years until reaching 3,737 cases. Reducing the scale of the scope of the data, in the state of Pará, this reduction occurred only in 2019 (Fig 1). In addition, the new Atlas of Violence, released in 2023 [9], indicates an estimate for rape cases in 2019. The authors estimate that the state of Pará is the largest, in the northern region of the country, in the occurrence of rapes with 42,232 cases. Of these, only 4.41% to 6.38% are registered.

According to the Brazilian Institute of Geography and Statistics (IBGE, in Portuguese) (2021), the State of Pará has 144 municipalities, divided into 21 immediate geographic regions, which are grouped into seven intermediate geographic regions. According to the Atlas of Violence, the state occupies the fourth position in homicides of women [8].

In this sense, this research aims to develop a multi-criteria decision analysis model (MCDA) to classify municipalities in Pará with regard to the propensity of the crime of VAW, based on the mapping of assistance and protection equipment, as well as socioeconomic indicators of each municipality.

This study represents an innovative approach to classifying municipalities in the state of Pará in terms of their risk of domestic violence against women. The differential of this research lies in the application of the ELECTRE Tri-B multi-criteria decision method, which allows for a comprehensive and weighted analysis of local socio-economic criteria and the availability of protection networks for women victims of domestic violence. In addition, this model is flexible and dynamic, allowing the criteria to be adapted as new indicators emerge and the possibility for experts to interact with the model to define personalised weights for each criterion.

This approach not only makes a significant contribution to the state of the art in the field of combating domestic violence against women, but also provides a vital tool for guiding strategic actions and public policies to address this serious social problem. By enabling the precise identification of the most vulnerable areas and the effective allocation of resources, this study has the potential to save lives and substantially improve the quality of life of women throughout the state of Pará.

## 2 Possible determinants of violence against women

Socioeconomic and demographic indicators are widely studied as factors that can contribute to VAW. According to data from the IBGE, black, poor, and poorly educated women are more likely to be victims of domestic violence in Brazil [8].

[10] shows that gender-based violence is a constant among adolescent girls and young adult women in low-income countries. In turn, lack of financial autonomy is a crucial factor in perpetuating domestic violence, since women who are financially dependent on their partners are less likely to be able to leave an abusive situation, which increases the risk of violence; in addition, women with precarious jobs and low wages also face difficulties leaving a violent relationship [11].

Thus, the level of schooling and access to education end up being factors of paramount importance within the context of VAW. According to [12], higher levels of education among women are generally considered protective factors against the risk of suffering domestic violence. In this sense, women with less education are more likely to experience physical, psychological, and sexual violence. This is due, in part, to a lack of resources to recognize and deal with abusive situations [13].

However, among the factors intertwined with level of education and income that represent conditioning factors of greater or lesser risk to women of being victims of violence, the demographic issue should not be overlooked; thus, women living in rural and remote areas face more difficulties in obtaining help in case of violence, as they often lack access to support and protection services. In addition, women belonging to ethnic or religious minorities face a combination of social inequalities that make them even more vulnerable to violence [14].

In the present work, we start from the consideration of the importance that these factors have for a greater or lesser incidence or risk of VAW; thus, regarding the municipalities and regions of Pará, we consider the indexes that, in our view, best represent the factors pointed out in the previous paragraphs given their importance from the socioeconomic point of view, namely: the Support and Protection Index (SPI), the Human Development Index (HDI), the Gross Domestic Product (GDP) per capita and the Gini Index, which help to understand the social and economic context in which domestic violence occurs.

HDI is an indicator that measures the human development of a country, taking into account factors such as life expectancy, education, and per capita income. The HDI can be useful for understanding the relationship between domestic violence and human development, since municipalities with lower HDI tend to have higher rates of VAW.

GDP per capita is a measure that indicates the average wealth of a population. Countries with a higher GDP per capita tend to have lower rates of VAW. This can be explained in part by the fact that women in wealthier countries have more resources to receive help and protection in the event of violence.

The Gini Index measures income inequality. Higher values tend to have higher rates of domestic violence, as income inequality can lead to greater economic and social vulnerability for women.

Similarly, the importance of support and protection services for women victims of violence must be recognized. Support and protection services are essential to help women overcome violence and resume their lives. These services may include specialized police stations, shelters, psychological support centers, legal, human rights, and social services. These services provide a safe environment for women victims of violence, offering them protection and support to deal with physical and psychological trauma caused by violence [15].

As described by [16], the network of women’s support services is critical to ensuring that victims receive adequate and effective assistance. These services should be accessible to all women, regardless of their geographic location or financial situation.

In Brazil, there are many support and protection services for women victims of violence, such as shelters, women’s service centers, specialized police stations, and halfway houses, among others. For example, Casa da Mulher Brasileira is an integrated center of services that offers psychological support, social assistance, legal services, and temporary shelter for women victims of violence.

However, access to these services can vary according to the region and the availability of resources. This is why the creation of a specific index to classify municipalities based on the quality of these services is an urgent need. Such an index could help identify areas where services are affordable and where more resources need to be invested to improve them.

In summary, these socioeconomic indicators are important for us to understand VAW and guide effective public policies to combat this problem. However, we must remember that domestic violence is a complex problem involving multiple factors and that it requires an interdisciplinary and multifaceted approach to be successfully combated. Additionally, it is essential to ensure that all women, regardless of their demographics, have access to the support and protection services necessary to live free from violence.

## 3 Related work

Currently, there are several solutions that apply computational techniques for the classification of regions in the context of violence. However, when it comes to multicriteria decision methods, there is a certain limitation in the scientific literature for problems of this nature; despite this, the authors [17] applied a multicriteria decision model combined with a geographic information system, with the objective of classifying zones of a given region, with higher rates of violence. The criteria used were, among others, demographic density, income concentration, and Human Development Index (HDI) of the researched region. The results proved to be different from a monocriteria analysis, demonstrating the importance of this methodology as a solution for the strategic management of problems of this nature.

In the work of [18], it is presented a multicriteria ELECTRE-Tri model that classifies municipalities of the state of Pernambuco, in the Northeast of Brazil, into categories according to their criminal characteristics. The author exemplifies actions to mitigate problems caused by criminal phenomena. After reviewing the literature, nine quantitative criteria were defined: vehicle theft, homicides, robbery of financial institutions, and bodily injury followed by death. In the results, the municipalities classified into four categories and the proposed guidelines for combining the most appropriate public security actions are presented.

Other multicriteria methods also solve classification problems such as the PROMSORT method, used by [19]. The work employs the multicriteria model for the classification of municipalities regarding crime, considering demographic and social factors such as: Gini index, per capita income, unemployment, demographic density, among others. The results clearly demonstrate the main areas of the state that have a high propensity to the occurrence of crime, as well as suggest strategic intervention in areas with higher crime indicators.

There is also the use of statistical methods used for the classification of the incidence of VAW cases risk, as observed in the work of [20], in which a spatial analysis is proposed, allowing the identification of the increase in the occurrence of VAW, as well as the areas of highest risk for such a phenomenon to occur. For the determination of risk, the authors assume that the probability of a phenomenon that occurred in the past is similar to the risk of that same phenomenon occurring in the future. Thus, they present risk maps of the researched areas, considered of great relevance for public administration, by offering the necessary support for decision making in the management of public resources.

In a recent work, [21] used a multicriteria decision method, the Analytic Hierarchy Process (AHP), to demonstrate the effectiveness of the application of this method regarding the implementation of public equipment to assist women victims of violence, specifically, the Casa da Mulher Brasileira (CMB). From the perspective of the authors and according to the research conducted, the criteria considered were the number of cases of violence in each of the five municipalities that make up the metropolitan region of Belém, capital of the state of Pará, the female population in each and their accessibility. The priority for the installation of the equipment was verified to be the city of Belém and, lastly, the municipality of Santa Bárbara do Pará.

In order to carry out evaluation of strategies to achieve feminicide reduction in Ecuador, [22] makes use of the neutrosophic TOPSIS technique. This made it possible to capture the expert evaluation criteria in a way that they were more in line with the truth, the ultimate purpose of the neutrosophic analysis. It was found that the most important decision alternative or strategy is to establish more preventive criminal legislation for the protection of victims of domestic violence.

Broadening the horizons on what is VAW, in [23] used geospatial technologies coupled with AHP to map potential zones of a very present sexual crime in India, namely, eve-teasing. Crime mapping with the help of Geographic Information System is a very useful tool for law enforcement agencies to make a proper visualization and take strategic modus operandi to reduce occurrences. The study also focuses on the decision making strategies of offenders to commit crimes. Such a study has larger implications for crime prevention in public places and improves the policy making process to alleviate such social nuisances.

Another aspect observed in the literature review was the validation of the proposed models. The authors [19, 20] consider the rates of complaints or occurrences as an important criterion for classifying a region; however, these rates could be used to validate the model, since the amount of complaints or occurrence records may depend on the access by society to public facilities that are generally located in large centers; in regions that lack these facilities, access to complaints is difficult.

Different from the works presented, this paper focuses on the proposal of a multicriteria classification model to present a mapping of Pará municipalities as to the propensity of VAW. For this classification, economic and social indexes will be analyzed in addition to quantitative characteristics of urban equipment in each municipality, considering the premises defined by [24]. This method will be validated later through the analysis of complaints, comparing the alternatives (municipality) with the record found. In addition and as a way to contribute to the scientific community, the source code for this work is available on the authors’ github repository. (https://github.com/jlucioDev/woman_victm_of_domestic_violence_ELECTRE_APP) In Table 1 we present a comparison of the works presented in this section with the model proposed in the paper.

**Table 1:** Comparison of related work.

	APPROACH	[17]	[18]	[19]	[20]	[21]	[22]	[23]	Proposed Model
1	Multicriteria methods support.	■	■	■	■	■	■	■	■
2	Geographic mapping of risk areas.	■	■	■	■	■	■	■	■
3	Use of public equipment indices.	■	■	■	■	■	■	■	■
4	Access to the model’s source code.	■	■	■	■	■	■	■	■
5	Validation based on complaints by zone.	■	■	■	■	■	■	■	■

■ Fully Meets ■ Partially Meets ■ Does Not Meet

## 4 Methodological procedures

The study is classified as exploratory due to the nature of the investigation: risk of VAW, considering public management scenarios and socioeconomic indicators of each municipality in Pará.

**Fig 2:**
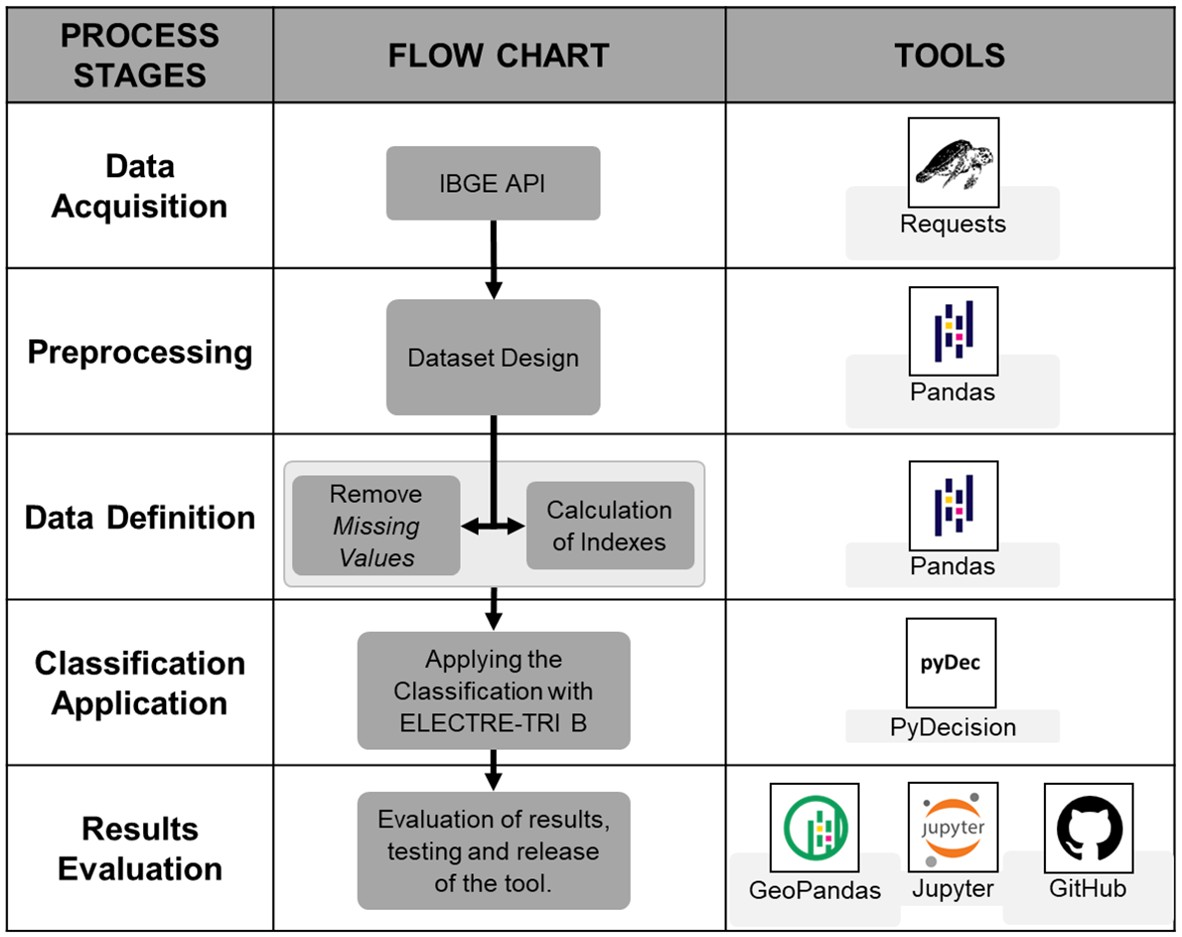
Project steps.

For the development of the study proposal, a series of steps was planned to allow the reproducibility of the model: 1. data acquisition; 2. pre-processing; 3. data treatment; 4. classification (ELECTRE-Tri B method); and 5. evaluation of the results. Fig 2 presents the five steps of the methodology and the tools used.

It is worth mentioning that the model chosen and used in this work can complement other already consolidated methods, such as statistical methods, once the multi-criteria models allow simultaneous evaluation of multiple variables.

### 4.1 Data acquisition

The data used in this study were acquired from the Brazilian Institute of Geography and Statistics (IBGE) via their data service API (https://servicodados.ibge.gov.br/api/v1). A substantial amount of data was obtained, which encompassed a wide range of sociodemographic and geographical indicators. The dataset is comprehensive, containing information from various regions and municipalities across Brazil.

The process of data retrieval involved a series of API requests to IBGE’s data service. These requests were tailored to collect specific socio-economic and demographic indicators relevant to the study of violence against women. Python, a versatile programming language, was used to streamline this data acquisition process.

Upon retrieving the data, the construction of the data set started. The raw data, as obtained from the API, underwent a rigorous data cleaning and processing phase. This included handling missing values, data normalization, and ensuring data integrity. The Pandas library (https://pandas.pydata.org/) was instrumental in performing these tasks efficiently.

The dataset encompasses an array of socio-economic indicators such as income distribution, education levels, and access to healthcare. These indicators are organized into matrices, enabling us to perform comprehensive analyses. The size of the matrices employed in this study is substantial, containing thousands of data points, making it possible to draw meaningful insights into the dynamics of violence against women.

To provide a clearer understanding of the acquired data, GeoPandas (https://geopandas.org/en/stable/) was used to create geospatial visualizations. These visual representations are crucial for geographically contextualizing the prevalence of violence and its correlation with socio-economic factors across different regions.

### 4.2 Pre-processing

For the comparative and classificatory analysis proposed by the work, it becomes important to define a data storage structure where each column corresponds to an indicator (criterion) and each row corresponds to the various regions analyzed (municipalities).

Then, after data consolidation, the dataset was built containing the information of each municipality according to the criteria described throughout this work.

### 4.3 Data treatment

Although the data collected and stored in the dataset can already be displayed, usually the values of different criteria are not comparable to each other, which makes it impossible to aggregate them immediately. To have them analyzed on the same scale, by the model, it is proposed to apply a normalization process, so that the resulting indicator value is between 0 and 1. For this, the linear variation proposed by [25].

$$\begin{array}{c} x_{i}=\frac{R_{i}-R_{min}}{R_{max}-R_{min}}xNormalizedinterval \end{array}$$ (1)

Where *R*_*i*_ is the criterion value to normalize and *R*_*max*_ and *R*_*min*_ are the maximum and minimum values of the criteria, respectively, and the range to adopt for normalization is, in general, between 0 and 1.

In only one case was it necessary to represent null values. These values refer to a municipality that did not provide the information to IBGE during the census. In this specific case, the value 0 (zero) was taken, which means that there is no resource for the indicator and, therefore, that it did not participate in the classification process.

#### 4.3.1 Measuring the level of adoption of protection and support mechanisms for women victims in the municipalities

The fight against VAW is carried out through an organized set of equipment that acts collaboratively in order to offer services and actions for the formation of a protection network. This network plays a fundamental role in combating violence and guaranteeing rights, as well as in the care and assistance of women victims of violence. This study considered protective and assistance equipment in municipalities, according to Table 2.

**Table 2:** Indicators that compose the SPI index.

INDICATOR—Equipment	Data Source
1. Existence of a police station specialized in serving women.	Instituto Brasileiro de Geografia e Estatística (IBGE)—Municipal Basic Information ResearchMunicipal MUNIC, year of 2019.
2. Executes programs and actions for women.	
3. Existence of campaigns against violence against women.	
4. Existence of rights or policies for women.	
5. Existence of policies or programs in the area of human rights– Protection of women victims of violence	

Thus, it was decided to determine an index of adoption of protection and assistance equipment in each municipality, aggregating the values of the five indicators, quantified by assigning scores. A score of 1 (one) was attributed to the existence of the equipment in the municipality, and a score of 0 (zero) was attributed to the absence of the equipment, with the lowest value being 0 and the highest value 5.

The Support and Protection Index (SPI) was first obtained by adding up the values of the five criteria. Then, in order to normalize the data, the variation defined by [25] was used, given by the division between the difference of the aggregated value (*V*) by the minimum value found (*V*_*min*_) and the difference of the maximum value obtained *V*_*max*_ and the minimum value *V*_*m*_*in*

$$\begin{array}{c} SPI=\sum_{i=1}^{5}i\frac{V-V_{min}}{V_{max}-V_{min}} \end{array}$$ (2)

Additionally, the same calculation will be used for normalization of the criteria later reported in this project.

### 4.4 ELECTRE TRI-B method

According to [26], multicriteria methods can be classified into three approaches according to the modeling of the decision maker’s preferences: single criterion synthesis approach, overclassification approach, and interactive local judgment approach. For this paper, the overclassification approach will be adopted.

The classification of the Pará municipalities as to the risk of violence is an important challenge for researchers and public administrators who work in the area of public security. For this, it is fundamental to choose a reliable and efficient classification method that can provide accurate and useful information for decision making.

To this end, the ELECTRE TRI-b method was chosen, which is able to handle imprecise and uncertain data, which is common in real ranking problems, [26, 27]. Another advantage is that this method is able to generate a ranking of the alternatives, which, compared to other ranking methods such as AHP and TOPSIS, offers specific advantages. AHP is a hierarchical analysis method that also takes into account multiple criteria, but it has the disadvantage of having to define a hierarchical structure for the criteria, which can be difficult in complex problems; TOPSIS is a method that uses the Euclidean distance between the alternatives and the criteria, which can be problematic in situations where the criteria are of different nature and cannot be directly compared.

According to [28], the ELECTRE Tri-B method was developed to solve ordered classification problems by comparing alternatives with reference profiles, which form the boundaries of each established class (category). Given that *G* = *g*_1_, *g*_2_, …, *g*_*j*_ is a set containing *j* criteria and each with a weight (level of importance) *w*_*j*_, for *X* = *x*_1_, *x*_2_, *x*_3_, … is a vector in *R*^*j*^ that represents the ratings of a generic alternative *x* on each criterion in *G*, and finally, whereas *B* = *b*_1_, *b*_2_, *b*_3_…, *b*_*n*_ is a set of *n* + 1 reference profiles where *b*_*h*−1_ and *b*_*h*_ are respectively the lower and upper bounds for the *h*^*th*^ class.

For each class *h* and each criterion *j*, *g*_*j*_(*b*_*h*_), represents the evaluation of the upper bound of *h*^*th*^ class by the *j*^*th*^ criterion. For each alternative *x*_*i*_ and each criterion *j*, *g*_*j*_(*x*_*i*_), represents the evaluation of *i*^*th*^ alternative for the *j*^*th*^ criterion. Overclassification depends on the absolute value of the difference *g*_*j*_(*x*_*i*_) − *g*_*j*_(*b*_*h*_), being greater than the predetermined thresholds of indifference (*q*_*j*_), preference (*p*_*j*_) and vector (*v*_*j*_), where *v*_*j*_ ≥ *p*_*j*_ ≥ *q*_*j*_. Then, the following steps are performed to obtain the overclassification relations.

**a**) Calculation of the degree of partial concordance *c*_*j*_(*x*_*i*_, *b*_*h*_) e *c*_*j*_(*b*_*h*_, *x*_*i*_) (Eqs 3 and 4)
  $$\begin{array}{c} c_{j}\left(x_{i},b_{h}\right)=\{\begin{array}{ccc} 0, & if & g_{j}\left(b_{h}\right)-g_{j}\left(x_{i}\right)\geq p_{j}\\ 1, & if & g_{j}\left(b_{h}\right)-g_{j}\left(x_{i}\right)<q_{j}\\ \frac{p_{j}-g_{j}\left(b_{q}\right)+g_{j}\left(x_{i}\right)}{p_{j}-q_{j}} & if & q_{j}\leq g_{j}\left(b_{h}\right)-g_{j}\left(x_{i}\right)<p_{j} \end{array} \end{array}$$ (3)
  $$\begin{array}{c} c_{j}\left(b_{h},x_{i}\right)=\{\begin{array}{ccc} 0, & if & g_{j}\left(b_{h}\right)-g_{j}\left(x_{i}\right)\geq p_{j}\\ 1, & if & g_{j}\left(b_{h}\right)-g_{j}\left(x_{i}\right)<q_{j}\\ \frac{p_{j}-g_{j}\left(x_{i}\right)+g_{j}\left(b_{q}\right)}{p_{j}-q_{j}} & if & q_{j}\leq g_{j}\left(x_{i}\right)-g_{j}\left(b_{h}\right)<p_{j} \end{array} \end{array}$$ (4)
**b**) Calculation of the global concordance degree *C*(*x*_*i*_, *b*_*h*_) e *C*(*b*_*h*_, *x*_*i*_) (Eqs 5 and 6)
  $$\begin{array}{c} C\left(x_{i},b_{h}\right)=\frac{\sum_{j=1}^{n}w_{j}c_{j}\left(x_{i},b_{h}\right)}{\sum_{j=1}^{n}w_{j}} \end{array}$$ (5)
  $$\begin{array}{c} C\left(b_{h},x_{i}\right)=\frac{\sum_{j=1}^{n}w_{j}c_{j}\left(b_{h},x_{i}\right)}{\sum_{j=1}^{n}w_{j}} \end{array}$$ (6)
**c**) Calculation of the degree of partial discordance Dj(xi, bh) and Dj(bh, xi) (Eqs 7 and 8)
  $$\begin{array}{c} D_{j}\left(x_{i},b_{h}\right)=\{\begin{array}{ccc} 0, & if & g_{j}\left(b_{h}\right)-g_{j}\left(x_{i}\right)<p_{j}\\ 1, & if & g_{j}\left(b_{h}\right)-g_{j}\left(x_{i}\right)\geq v_{j}\\ \frac{-p_{j}-g_{j}\left(x_{i}\right)+g_{j}}{v_{j}-p_{j}} & if & p_{j}\leq g_{j}\left(b_{h}\right)-g_{j}\left(x_{i}\right)<v_{j} \end{array} \end{array}$$ (7)
  $$\begin{array}{c} D_{j}\left(b_{h},x_{i}\right)=\{\begin{array}{ccc} 0, & if & g_{j}\left(x_{i}\right)-g_{j}\left(b_{h}\right)<p_{j}\\ 1, & if & g_{j}\left(x_{i}\right)-g_{j}\left(b_{h}\right)\geq v_{j}\\ \frac{-p_{j}-g_{j}\left(b_{q}\right)+g_{j}}{v_{j}-p_{j}} & if & p_{j}\leq g_{j}\left(x_{i}\right)-g_{j}\left(b_{h}\right)<v_{j} \end{array} \end{array}$$ (8)
**d**) Calculation of the degree of credibility *σ*(*x*_*i*_, *b*_*h*_) e *σ*(*b*_*h*_, *x*_*i*_), that expresses confidence in the expression “*x*_*i*_ is no worse than *b*_*h*_” (Eqs 9 and 10).
  $$\begin{array}{c} \sigma \left(x_{i},b_{h}\right)=\{\begin{array}{ccc} C\left(x_{i},b_{h}\right)\times \prod_{j=1}^{n}\frac{1-D_{j}\left(x_{i},b_{h}\right)}{1-C(x_{i},b_{h})}, & if & D_{j}\left(x_{i},b_{h}\right)>C\left(x_{i},b_{h}\right)\\ C(x_{i},b_{h}), & else \end{array} \end{array}$$ (9)
  $$\begin{array}{c} \sigma \left(b_{h},x_{i}\right)=\{\begin{array}{ccc} C\left(b_{h},x_{i}\right)\times \prod_{j=1}^{n}\frac{1-D_{j}\left(b_{h},x_{i}\right)}{1-C(b_{h},x_{i})}, & if & D_{j}\left(b_{h},x_{i}\right)>C\left(b_{h},x_{i}\right)\\ C(b_{h},x_{i}), & else \end{array} \end{array}$$ (10)
**e**) The overclassification decision still depends on a previously determined parameter, the lambda cut-off level (λ) (Eq 11).
  $$\begin{array}{c} \begin{array}{cccc} x_{i}Sb_{h}, & \;Se & \sigma (x_{i},b_{h})\geq \lambda ; & \;0.5\leq \lambda \leq 1 \end{array} \end{array}$$ (11)

### 4.5 Classification

Since it was necessary to use socioeconomic indicators that characterize the municipalities and regions in order to have a common and acceptable basis in the literature, in this research we adopted the four indicators, namely: Support and Protection Index (SPI), Human Development Index (HDI), Gini Index [29] and the Gross Domestic Product (GDP) that was chosen arbitrarily by the decision maker. Table 3 shows the description of all the criteria evaluated.

**Table 3:** Evaluation criteria.

CODE	CRITERIA	DEFINITION	MAX/MIN
C1	SPI (Support and Protection Index)	Calculated by aggregating the values of the existence or not of victim assistance and protection agencies in the municipality.	Maximize
C2	HDI (Human Development Index)	It is a comparative indicator used to segment developed, developing and underdeveloped countries.	Maximize
C3	GDP per capita	It is the gross domestic product divided by the number of inhabitants	Maximize
C4	GINI (Gini Index)	It measures the degree of income concentration within a given group. It shows the difference between the incomes of the poorest and the richest (IPEA, 2019).	Maximize

The model was applied to the 144 municipalities of the state of Pará, located in the Brazilian Amazon region. The data were extracted from the Municipal Basic Information Research (MUNIC 2019). The aforementioned survey provides, among other information, the structure of municipal public management, including the existence of urban protection equipment, programs or actions to combat violence, and specific legislation to confront VAW.

The initial parameters were defined from the dataset analysis, by calculating the median. Table 4 presents the values of the class limits (*b*_*h*_) for each criterion. The definition of five limits characterizes the distribution of the alternatives in six classes. To determine the ranking of the alternatives (municipalities), the multicriteria analysis and decision method ELECTRE TRI-B was used. This method was developed to solve classification problems by comparing the alternatives with reference profiles, which form the boundaries of each class [28].

**Table 4:** Limit values for each criterion.

LIMIT PROFILES	CRITERIA			
	SPI	HDI	GDP per capita	GINI
b1	0.1	0.48	0.01	0.37
b2	0.2	0.57	0.03	0.68
b3	0.3	0.62	0.04	0.70
b4	0.4	0.68	0.08	0.72
b5	0.5	0.69	0.50	0.85

As observed in Table 5, for the weight values *w*_*j*_, it was considered the HDI and Gini criteria with higher values, followed by the SPI and GDP criteria. For the preference values *q*_*j*_ and *p*_*j*_ and indifference *v*_*j*_, it was considered the analysis of the scores of each criterion.

**Table 5:** Preference and indifference values.

Parameters	CRITERIA			
	SPI	HDI	GDP per capita	GINI
**q**	0.1	0.10	0.1	0.1
**p**	0.2	0.20	0.2	0.2
**v**	1.0	1.00	1.0	1.0
**w**	0.5	1.00	0.3	1.0

Initially, a cut level λ = 0.60 was defined and for the sensitivity analysis making adjustments of 0.05 for plus and minus, considering the same weights. Then the weights of the criteria were adjusted by selecting each one and setting a value of 1.00 and the others 0.50. However, no changes were identified in the results, thus concluding that the model is robust and shows good sensitivity performance.

## 5 Results

In this section, details of the results of applying the model using the multi-criteria method are presented.

After running the model, the data presented showed the municipalities classified into six categories with regard to the propensity of VAW cases: Very Low; Low; Medium; High; Very High; Extremely High.

Table 6 presents the distribution of municipalities in their respective categories. Quantitatively most of the municipalities were classified in the Very High category. This indicates the regions most lacking in public resources for the protection of rights.

**Table 6:** Result of the municipalities classification.

**Very Low**	Almeirim; Barcarena; Canaã dos Carajás; Capanema; Castanhal; Marabá; Tucuruí
**Low**	Ananindeua; Belém; Conceição do Araguaia; Marituba; Paragominas; Parauapebas; Santarém; Vitória do Xingu
**Medium**	Abaetetuba; Altamira; Itaituba; Juruti; Mãe do Rio; Nova Ipixuna;Óbidos; Oriximiná; Redenção; Rondon do Pará; São Félix do Xingu; São Geraldo do Araguaia; Soure; Tailândia
**High**	Benevides; Bom Jesus do Tocantins; Brasil Novo; Bujaru; Cachoeira do Arari; Cametá; Capitão Poço; Colares; Concórdia do Pará; Cumaru do Norte; Curionópolis; Eldorado do Carajás; Inhangapi; Jacundá; Maracanã; Mocajuba; Monte Alegre; Muaná; Nova Timboteua; Novo Repartimento; Ourém; Pau D’Arco; Primavera; Salinópolis; Santarém Novo; São Caetano de Odivelas; São Domingos do Araguaia; São Miguel do Guamá; Tomé-Açu; Xinguara
**Very High**	Abel Figueiredo; Acará; Afuá; Água Azul do Norte; Alenquer; Anajás; Anapu; Augusto Corrêa; Aurora do Pará; Aveiro; Bagre; Baião; Bannach; Belterra; Bonito; Bragança; Brejo Grande do Araguaia; Breu Branco; Breves; Cachoeira do Piriá; Chaves; Curralinho;Curuá; Curuçá; Dom Eliseu; Faro; Floresta do Araguaia; Garrafão do Norte; Goianésia do Pará; Gurupá; Igarapé-Açu; Igarapé-Miri; Ipixuna do Pará; Irituia; Itupiranga; Jacareacanga; Limoeiro do Ajuru; Magalhães Barata; Marapanim; Medicilândia; Moju; Nova Esperança do Piriá; Novo Progresso; Oeiras do Pará; Ourilândia do Norte; Pacajá; Palestina do Pará; Peixe-Boi; Piçarra; Placas; Ponta de Pedras; Portel; Porto de Moz; Prainha; Quatipuru; Rio Maria; Rurópolis; Salvaterra; Santa Bárbara do Pará; Santa Cruz do Arari; Santa Izabel do Pará; Santa Luzia do Pará; Santa Maria das Barreiras; Santa Maria do Pará; Santana do Araguaia; Santo Antônio do Tauá; São Domingos do Capim; São Francisco do Pará; São João da Ponta; São João de Pirabas; São João do Araguaia; São Sebastião da Boa Vista; Sapucaia; Senador José Porfírio; Terra Alta; Terra Santa; Tracuateua; Trairão; Tucumã; Ulianópolis; Uruará; Vigia; Viseu
**Extremely High**	Melgaço

**Fig 3:**
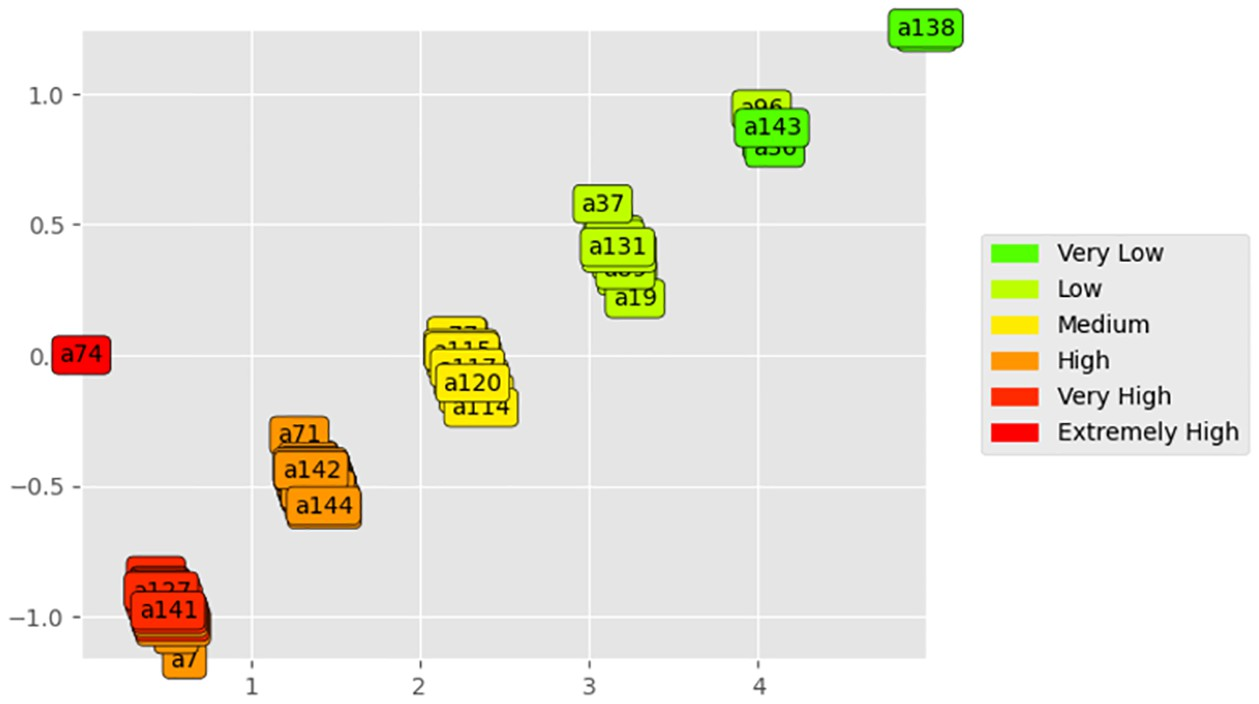
Class distribution.

Fig 3 represents the result of running the model using the PyDecision library. The graph shows the distribution after the overclassification results in the final evaluation of the criteria and class thresholds. In this section, the details of the results of the model application using the multicriteria method are presented.

**Fig 4:**
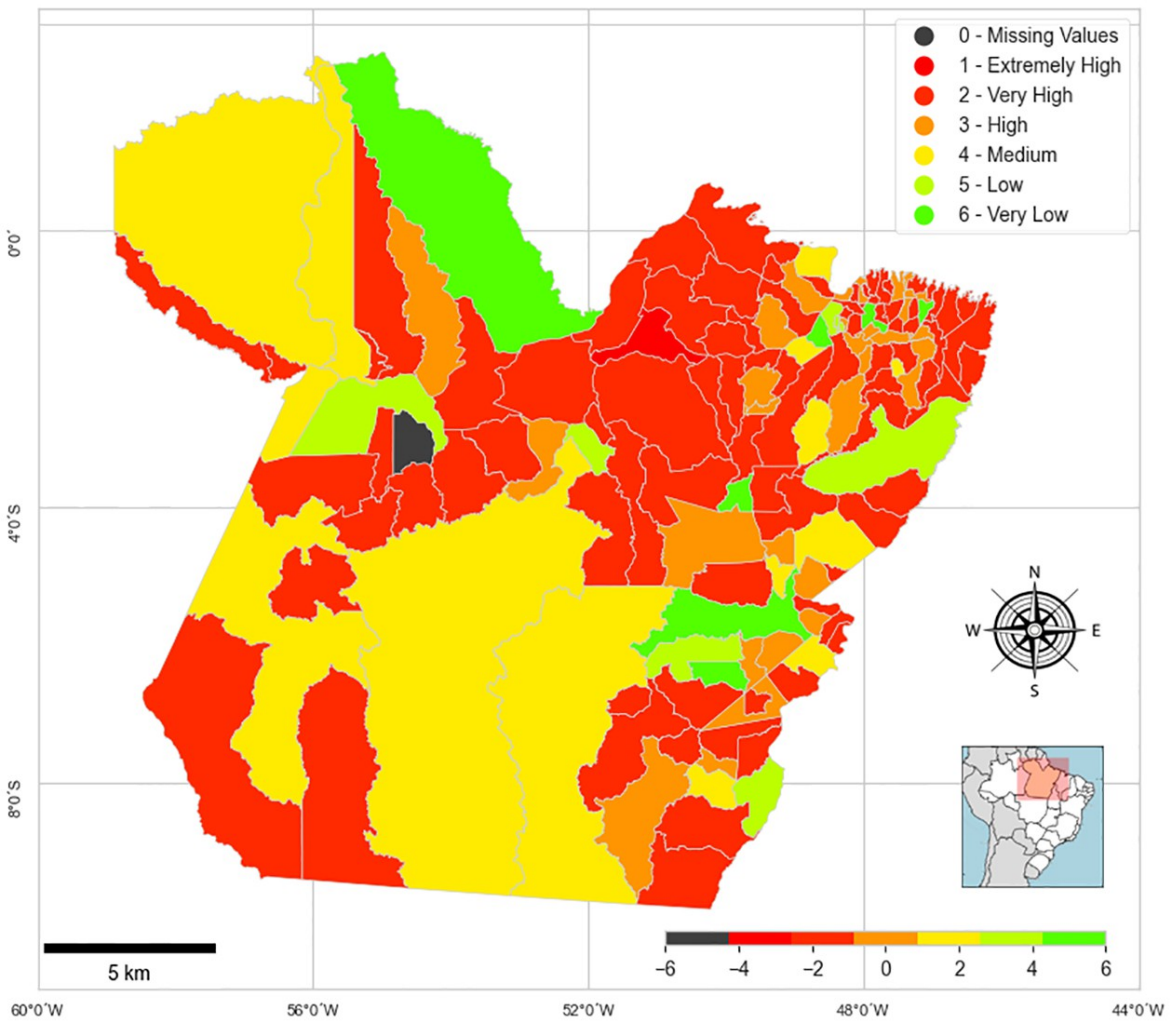
Geographical mapping as to propensity to violence. This figure was generated using the Geopandas [30] (https://geopandas.org) and Python Programming Language (version 3.10). The underlying basemap is derived from ‘Natural Earth’ global vector data (https://www.naturalearthdata.com).

In the analysis of the mapping of municipalities, as shown in Fig 4, it is possible to identify that the regions that have very low propensity (areas in green) are distributed between the areas of the lower Amazon and southeastern Pará. It is also important to notice that the regions of southwest Pará were mostly classified between medium and high risk, thus being characterized as areas lacking resources and, therefore, more in need of State interventions.

Also noteworthy are the Melgaço municipality classified as extremely high the propensity of VAW cases. The municipality of Melgaço has low socioeconomic indicators and only one equipment for protection and assistance to women victims. In the case of Mojuí do Campos, the indicators appear with a value of zero due to the municipality’s refusal to provide information, according to IBGE data. For this reason, only this municipality did not participate in the classification.

**Fig 5:**
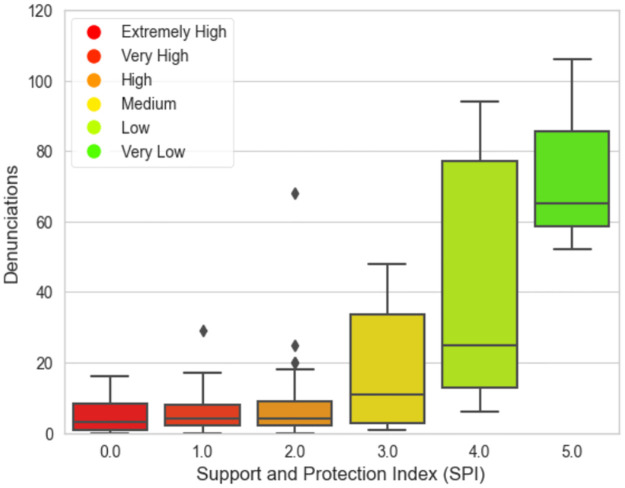
Denunciations—Women’s call center, Ligue 180.

As referential data in this work, the quantities of denunciations were aggregated through the analysis of data obtained from the Ministry of Women’s website (https://www.gov.br/mdh/pt-br/acesso-a-informacao/dados-abertos/ligue180) and inserted into the original dataset in order to verify whether the quantity of protection and assistance equipment, as well as the socioeconomic indicators, are related to the rate of denunciations in each region. In Fig 5 are shown the records of the Denunciations of the Ligue 180.

However, in municipalities classified as very low or low risk it was noticed the considerable existence of protection equipment, good socioeconomic indicators, but a high rate of denunciations. Given this, [24] conclude that this result can be explained by the fact that environments with more protection mechanisms for women—that is, a greater number of shelter homes and support centers—provide a greater number of reports of aggression. Thus, it is through the presence of such protection mechanisms that society’s disapproval and women’s sense of security is increased, thus enabling the disappearance of reports.

## 6 Discussion

The goal set for this work was to classify municipalities in the state of Pará with regard to the probability of VAW occurring or not; to do so, a multicriteria approach was developed based on the ELECTRE TRI-b method and, based on this, socioeconomic factors represented by the indexes described throughout this article were considered as references, besides keeping in mind the existence or not of support equipment for women, especially those who have been victimized, but also public equipment and services that serve as encouragement for more complaints to be made.

Thus, our goal, after this course, is to establish a mapping of municipalities and regions with more and less propensity to be territories where the VAW is rife and, even so, do not have specific public policies to put an end to or mitigate this problem; having said this, it is now up to us to establish the correlations between our research and the context in which it is located and where the results achieved fit in.

From the point of view of the application of the method from the referential indexes, it can be seen that it was able to provide satisfactory results supporting a classification of municipalities that, in part, can corroborate the empirical experiences of the authors, but, mainly, confirm what the official data have; Thus, considering, for example, the municipalities classified as “Very Low” risk for VAW, we have a group of federal units with good or excellent socioeconomic development indexes, as is the case of the city of Canaã dos Carajás, one of the highest GDPs in the state of Pará.

This does not mean that good or excellent socioeconomic development indexes are the causes for the low possibility of VAW in its territory; it does mean that the presence of public services and equipment to assist women and the relative development it experiences may be combining to reduce the cases of VAW.

This can be contrasted with, for example, the municipality of Melgaço; this is one of the municipalities with the lowest development indexes in the state of Pará and in the whole Amazon region, in addition to being far from urban centers and still having little or no presence of public equipment and services to support women; this combination can be the root for higher risks of VAW in its territory.

Such correlations can also be made when taking the data from the Brazilian Federal Government’s Ligue 180 (https://www.gov.br/mdh/pt-br/acesso-a-informacao/dados-abertos/ligue180). It can be seen that the more central the municipality is, the higher the number of calls to this service, which is available to receive reports of VAW throughout the country. If it is true that we cannot state definitively that the data from Ligue 180 corroborate the findings of this research, it is equally true that they show important overlaps since they reveal approximations with, for example, the fact that the three largest and most central municipalities in the State (Belém, Ananindeua, and Santarém) appear with a “Low” risk classification for VAW. The first two are in the metropolitan region of the state, while the third is in the western region; having a relatively good network of equipment and services, but not having the best socio-economic indexes of the state, these municipalities have a better network of information and devices favorable to accusations, which can explain the greater action of the defense and protection agencies.

Therefore, the mapping prepared at the end of this research can be a valuable tool for public agencies and other institutions that work in the sphere of defense and protection of women victims of VAW or that are under imminent risk; knowing where the points that deserve more attention are is already a good start for the formulation of public policies aimed at alleviating this problem, such as, for example, the implementation of campaigns or the implantation of public equipment and services, whether in the assistance sphere, or in the police, medical, or judicial sphere, in addition to those aimed at generating employment and income opportunities for women victims or that, being in abusive relationships, may feel encouraged to leave such relationship.

## 7 Conclusion

This article presents a multicriteria model developed to classify the municipalities of the State of Pará on the propensity of the existence of a favorable or not context for VAW; the dimensions used for this classification were the political, social, economic, and demographic scenarios. The ELECTRE TRI-B was used as a means to reach the objective.

The model developed and the research findings represent an important step for the elaboration, execution, and control of public policies better coordinated with the reality of each municipality in Pará; based on these data, institutions of public power and society can intervene in a proactive and preventive way regarding VAW.

On the other hand, the elaboration of a mapping, which can be constantly updated, is important in that it is a more didactic and replicable instrument for monitoring the VAW propensity reality in other situations and locations, given the efficiency of the model proposed here.

Furthermore, regarding the choice of the multi-criteria method in relation to other approaches that use statistical methods, we can say that multi-criteria models allow for the simultaneous evaluation of multiple criteria, which is important when we are dealing with a complex set of data that cannot be easily summarized into a single variable. Moreover, these models can handle data of different types and scales, which is particularly useful when working with heterogeneous data.

In summary, it is believed that the multi-criteria approach was an appropriate choice for this study, given the complex set of data being dealt with and the possibility of involving stakeholders in the decision-making process. However, it is recognized that there are several methodological options available that could be compared, in future work, with the methodology proposed here.

In the final analysis, the originality of this study lies in its ability to provide a comprehensive and adaptable approach to understanding and preventing violence against women at the municipal level. We believe that our findings not only fill a gap in existing research, but also offer a valuable perspective that can inform concrete strategies to tackle gender-based violence. We hope that this work will inspire researchers and policymakers to consider multifaceted and dynamic approaches to solving this critical challenge and thus contribute to a safer and more equal world for all women.

We believe that violence against women is a complex and multifaceted phenomenon that cannot be fully understood through a single set of social determinants. Our study, by focusing on socio-economic indicators, allowed us to analyze a significant part of this puzzle, but we are aware that gender-based violence transcends the boundaries of social class and education. In many cases, violence persists in communities and families with higher levels of education and standard of living, hidden under the veil of social stigma and established patriarchal structures. We recognise the importance of a more in-depth analysis of these cultural contexts and values that perpetuate violence, and we encourage future research to explore this critical dimension.

This study represents a significant step forward in understanding violence against women, but we also recognize that it is only one aspect of a much larger problem. Our research highlights the interconnection between social determinants, inequality, and gender-based violence, but does not claim to be the definitive answer. Instead, we hope that this work will stimulate an ongoing critical dialogue about the nature of violence against women, its manifestation in diverse communities, and the challenges of combating it effectively. As we move forward, it is imperative that we consider not only the quantitative factors, but also the cultural and social aspects that perpetuate violence, in order to develop more holistic and context-sensitive strategies. Together, we can work to create an environment in which all women, regardless of their social background, live free from the shadow of violence.

We hope that further research can deepen our understanding of the underlying complexities, including analyzing the cultural and social dynamics that perpetuate violence. In addition, we encourage studies that explore specific intervention strategies, targeted public policies, and innovative approaches to prevention and victim support. As we continue to expand our knowledge of this critical issue, we are confident that we can work toward a future in which VAW is an increasingly rare and unacceptable reality.
